# Development of a Group Emergent Literacy Screening Tool

**DOI:** 10.3390/children10020306

**Published:** 2023-02-06

**Authors:** Joana Cruz, Sofia Mendes, Sofia Marques, Diana Alves, Irene Cadime

**Affiliations:** 1 Centro de Investigação em Psicologia para o Desenvolvimento, Instituto de Psicologia e Ciências da Educação, Universidade Lusíada Porto, 4100-346 Porto, Portugal; 2 Centro de Psicologia da Universidade do Porto, Faculdade de Psicologia e Ciências da Educação da, Universidade do Porto, 4200-135 Porto, Portugal; 3 Centro de Investigação em Psicologia, Escola de Psicologia, Universidade do Minho, 4704-553 Braga, Portugal

**Keywords:** emergent literacy, screening tool, Rasch model, validity, reliability

## Abstract

It is important to identify children who are struggling with emergent literacy skills as early as possible to provide them with the support they need to prevent future academic failure. Screening tools administered in groups are more cost-effective than those administered individually, but few are available in Portugal. The goal of this study was to explore the psychometric properties (difficulty, reliability, and validity) of a group emergent literacy screening test for Portuguese-speaking children. The test includes two phonological awareness tasks, one vocabulary task, and one concepts of print task. The sample comprised 1379 children from pre-K (*n* = 314), kindergarten (*n* = 579), and first grade of primary education (*n* = 486). Measures of emergent literacy, reading and writing skills, and academic achievement were used to test the validity of the screening test. The Rasch model results suggest that the tasks were suitably difficult for the kindergarten group, but had varying levels of difficulty for pre-K and first grade. Reliability was adequate for the tasks with an appropriate level of difficulty. Scores for the screening test were highly correlated with measures of literacy and with academic achievement. These findings suggest that the presented emergent literacy screening test is valid and reliable, making it a useful tool for practice and research.

## 1. Introduction

Promoting emergent literacy has become a priority for educators, researchers, and policymakers [1,2] due to evidence demonstrating the importance of these skills for future success in reading and writing [3,4,5,6]. Since Mary Clay [7,8] pioneered the analysis of young children’s conceptions of reading and writing when researching language acquisition, emergent literacy framework has grown considerably. Clay [7] analyzed in detail how children acquired written language and how this acquisition progressed due to the interactions with adults and with written materials. These studies lead her to reject the concept of “maturity for reading and writing”, considering that it does not capture the dynamics of an active process of acquisition and extraction of rules regarding the relationship between oral and written language. However, in Portugal, there has not been much systematic effort to identify children who may be at risk for literacy difficulties during the preschool years.

The “wait-to-fail” model [9] has been prevalent for a long time in the Portuguese education system. Consequently, many children who are at risk of difficulties, but who do not exhibit significant “red flags”, go undetected until there is a severe reading or writing problem [10,11]. Nevertheless, there has recently been a shift in Portuguese educational policies towards a new framework that emphasizes the need for equity and inclusion for all students [12,13] through multitier systems of support, early identification, and timely intervention. This conceptual framework aims to ensure that each student has the best opportunities for academic success, allows for the identification of children who are at risk, monitors the progress of all children to adjust the type, intensity, and frequency of intervention, and emphasizes that each child should receive evidence-based instruction and the implementation of levels of support considering the child’s response to the intervention [14].

In preschool settings, meeting the needs of all children can be challenging due to the diversity in linguistic and early literacy skills among children [15]. Another challenge is identifying children who may need and benefit from supplemental intervention before they begin receiving formal reading and writing instruction. The goal of this study is to evaluate the psychometric properties of a Portuguese emergent literacy screening tool designed for group administration, intending to identify children with poor emergent literacy skills.

### 1.1. The Need for Early Identification

Research shows the importance of providing tools for the early identification of children who may be at risk of reading and writing difficulties [1,16]. Screening procedures used during the preschool years can help identify children who may need monitoring, as well as identify their areas of weakness, and thus can be used to help plan proper preventive interventions to improve those skills from as young as age 3 or 4 [1,17,18]. This can facilitate their learning and development [19].

Evidence regarding intervention in emergent literacy skills before the onset of formal reading and writing instruction supports the relevance of the Response to Intervention (RtI) model and the need to implement this framework for preventing learning disabilities [2]. This tiered system of support can help to identify children who may require additional intervention and determine the appropriate levels of intervention to increase the chances of success for all children [6,13]. Under the RtI model, universal screening can be utilized to plan, modify, and tailor instruction based on evaluation data [20]. It can also serve as a data-driven guide for identifying children who may need a more intensive response-based intervention [2], and those who are likely to succeed [4]. Universal screening involves evaluating all children in a group, grade, or school and predicting their risk status, helping deliberate interventions to prevent reading failure and decrease the number of children identified with specific learning disabilities [21].

### 1.2. The Assessment of Emergent Literacy Skills

The number of available screening tools for measuring emergent literacy skills is generally increasing in most countries [2,6,13,16,17,22,23]. Usually, these tools integrate brief, easy-to-use measures with clear scoring and interpretation, have a low cost, and provide information about the children’s performance in emergent literacy skills [12,13]. The results of such screenings are interpreted relative to at least one threshold, allowing users to make decisions on whether a child requires additional assessment or support interventions [1,21]. For example, Ecalle and colleagues [1] proposed a threshold of one standard deviation below the mean for each skill and recommended that low scores in two or more skills simultaneously may be an indicator of future difficulties in learning to read. Stuckey and Albritton [6] also used scores expressed as standard deviation units (Z scores) to identify students at risk in oral language. Specifically, they recommended providing Tier 1 interventions to students with Z scores >−1 (no risk), Tier 2 interventions to students with scores between one and two standard deviations below the mean, and Tier 3 interventions to students with scores two standard deviations or more below the mean. Ford and colleagues [4] suggested that thresholds can be used to identify children whose scores are at or above a certain level, in addition to detecting children at risk for reading difficulties.

The tools used to screen emergent literacy skills often assess multiple skills that predict success in reading [1,4,24]. Emergent literacy is a wide construct that comprises conceptual knowledge and procedural knowledge about reading and writing, as well as oral language and metalinguistic skills [25]. To develop emergent literacy skills, preschool children need to be supported by adults in their regular environment, and in a rich and stimulating context [26]. School and family should encourage children to acquire skills and serve as models, namely when they read books to children and interact with them regarding knowledge of written language, while sharing books, introducing different written materials (books, tickets, invitations, food menus, recipes, and so on), preparing shopping lists with children, and taking children to the library or bookstore [27].

Due to the multi-dimensional nature of oral language, researchers argue for the need to evaluate various language skills [11,14], such as phonological awareness and vocabulary [1,19,28]. Vocabulary is considered a key domain in emergent literacy research [1,14]. Dickinson and colleagues [3] suggest that vocabulary reflects a child’s effective use of language. Cabell and colleagues [29] also note that children who perform poorly in early literacy, but have strong oral language skills, may be more likely to benefit from whole-classroom instruction. Code-related skills including alphabet and letter knowledge [1,19,29], concepts about print [28], name writing [16,22], and emergent writing or basic writing skills [13] should also be evaluated during screenings because they are independent predictors of later reading and writing success and develop during preschool [1,6]. Meyer and colleagues [22] point out that oral language directly impacts code-related skills, such as phonological awareness, which contributes to understanding the alphabetic principle. Skills such as vocabulary, phonological awareness, concepts about print, and emergent writing do not develop independently of one another in preschool years; inter-correlations exist between oral and written language due to the intentional and systematic practices that occur in preschool settings [30]. Literature enhances the need to develop explicit, deliberate, and systematic training of these skills, because for some children, random or occasional stimuli do not promote emergent literacy growth [14,25,27].

Several studies evidence that oral language (namely vocabulary), print concepts, and phonological awareness at preschool might predict reading accuracy and reading comprehension from first to fourth grade [31,32,33]. A study with 34 children [31] assessed at the end of kindergarten and at the end of first grade showed through hierarchical multiple regression analyses that reading skills are mainly predicted by phonological awareness measured at the kindergarten stage and, subsequently, by phonological memory abilities measured at the end of first grade. Another study [33] examined code-related and oral language precursors to reading in a longitudinal study of 626 children from preschool through 4th grade. Structural equation modeling demonstrated that during early elementary school, reading ability is predominantly determined by the level of print knowledge and phonological awareness a child brings from kindergarten, and in later elementary school, reading accuracy and reading comprehension appear to be two separate abilities that are influenced by different sets of skills.

### 1.3. Screening Tools for Emergent Literacy Skills

There are several available emergent literacy screening tools for children who speak different languages (e.g., English, Spanish, and French). The Brief Early Literacy Screener, for example, assesses young English-speaking children’s emergent literacy skills, including alphabet knowledge, concepts about print, phonological awareness, and phonemic knowledge. It consists of 25 items in which the examiner reads the question at the top of the page and the child points to one of four pictures as the answer. Another English-language tool is the 10-item Early Literacy Skills Assessment Tool (ELSAT), which uses a shared book interaction [18] to evaluate print concepts, word awareness, letter knowledge, and phonological awareness. It can be completed in 1–2 min and requires minimal training. The Phonological Awareness Literacy Screening in Spanish for Preschool [22] consists of nine tasks to assess various oral language and code-related skills in Spanish-speaking children, including language production, narrative skills, listening comprehension, phonological awareness (syllable clapping, rhyme awareness, beginning sound awareness), alphabet knowledge, name writing, and concepts about print. Another Spanish screening test is the IPAL (Indicadores de Progreso de Aprendizaje en Lectura, or Indicators of Basic Early Reading Skills). The kindergarten sub-tests of the IPAL [23] include measures of letter-name fluency (naming the letter), letter-sound fluency (saying the sound of the letter), phonemic awareness, concepts about print (through a storybook and images), expressive vocabulary, and oral comprehension [2]. However, these four tools are administered individually, which takes a significant amount of time, leaving less time and resources available for providing intervention and support to children who need it [11].

Few emergent literacy screenings have a collective format, which can reduce costs in terms of time. One example of a collective screening test is the French Brief Screening Tool for Literacy Skills in Preschool Children [1]. Children respond to the items in four tasks presented in a booklet, which assess three domains: letter knowledge, phonological skills, and vocabulary. The letter-name knowledge task asks children to circle the letter (among seven) named by the teacher. Ten letters are selected, five with high frequency (G, P, D, C, R) and five with low frequency (J, V, Q, T, B). The phonological skills tasks include an auditory task where the teacher names three pictured words that contain a common syllable or phoneme (three items) and the children must circle the picture name that does not share a common unit. The second task is a syllable deletion task where children must retrieve a new pictured word after deleting the first syllable of a first word. The vocabulary task asks children to circle the picture (out of four) that corresponds to the word named by the teacher. Ten words are presented, five with high frequency and five with low frequency. According to Hendricks and colleagues [11], screeners that are administered to all children in a class can increase efficiency, maximize personnel resources, and minimize disruption to class time.

### 1.4. The Present Study

In some countries, preschool screening in literacy development has received little attention [16], or there is a lack of clarity about optimal early literacy screening measures to identify children for additional instructional support [6]. In Portugal, screening tools for emergent literacy skills in preschool children are scarce—although there are several assessment batteries to examine language domains and cognitive and motor development in an individualized format [34,35,36]. In terms of group screening tests, Batalha and colleagues [37] recently published a collective assessment instrument that aims to diagnose deficits in oral language, reading, and writing in children attending preschool (5 years) or the early years of primary school (first and second grades). This tool evaluates a broad range of skills, such as emergent literacy and decoding skills, but does not include vocabulary. Other early literacy screening tests, such as the RaLEPE [36], are also available in Portugal, but are not administered directly to children, instead being completed by parents/caregivers.

Therefore, a screening tool for 4–6-year-old Portuguese-speaking children was developed considering three domains that are known to be early literacy predictors of reading and writing: phonological awareness, vocabulary, and print knowledge [28,31,33,35]. This is a brief measure that was designed to be administered to children in groups, meaning it can be used in universal screenings with low costs before formal schooling begins, that follows the Portuguese Orientations for Preschool Education [38] and the evaluation guidelines for preschool education [39]. The goal of this study was to investigate the difficulty level and reliability of this measure and to collect evidence of validity based on the relationship with other variables.

## 2. Materials and Methods

### 2.1. Participants and Procedures

The study was approved by the ethics committee of the Psychology for Positive Development Research Center (CIPD/2122/DEED/1). Legal authorizations for data collection were also obtained from the Portuguese Ministry of Education (MIME—0570600007), school boards, and parents of participants. An informed consent for participation was requested of parents, according to the Declaration of Helsinki and the Oviedo Convention.

The sample consisted of 1379 children: 314 (22.8%) attended pre-K (aged 3–4 years old, mean age = 3.86, std. dev. = 0.348), 579 attended kindergarten (aged 4–6 years old, mean age = 4.87, std. dev. = 0.338), and 486 attended the first grade of primary school (aged 5–8 years old, mean age = 6.03, std. dev. = 0.366). All schools were public schools that were located in the north (N = 1253, 90.9%), center (N = 57, 4.1%), and south (N = 69, 5.0%) of Portugal. In all three age groups, boys and girls were represented approximately equally (see Table 1).

For pre-K and kindergarten children the screening tool was administered in small groups (four children). For first-grade children, the tool was administered in larger groups (classes). Data collection occurred in the first months of the school year (October and November 2021). The screening tool had no time limit for completion. After being administered the screening tool, subsamples of pre-K (*n* = 29), kindergarten (*n* = 55) and first-grade students (*n* = 40) were individually administered a set of standardized measures of emergent literacy and language that assessed phonological awareness, vocabulary (breadth and depth), and concepts about print. The first-grade subsample was also administered a letter recognition task. At the end of the school year (May and June 2022), the first-grade students were also administered standardized measures of word recognition and word writing. For these students, the final grades in the subject “Portuguese” and teachers’ ratings on each student’s reading and writing skills were also collected. Trained psychologists at the children’s schools administered all measures.

### 2.2. Measures

The Emergent Literacy Skills Universal Screening Test (DUCLE—Despiste Universal de Competências de Literacia Emergente). This test includes four tasks presented in a booklet for each child. The four tasks assess three emergent literacy domains: phonological awareness (two tasks), vocabulary (one task), and concepts about print (one task). Each task is preceded by a training item and is as follows:

Task 1: Phonological awareness (Initial syllable)—“Discover the initial sound”. This task has 13 items, each for which the examiner names four pictured words. Children have to circle the two pictures that represent words with a common starting unit (e.g., rato, raquete).

Task 2: Phonological awareness (Final syllable)—“Discover words that rhyme”. This task has 11 items. Children need to identify words with the same syllabic ending sounds. For each item, a target image is presented alongside three other pictured words. Children are invited to find the other word that has the same syllabic ending sound as the target word (e.g., João, balão).

Task 3: Vocabulary—“Where is it?”. Children are presented with four pictures and asked to circle the one that corresponds to the word named by the examiner (e.g., for the target word vaca [cow], the other pictured words are gato [cat], porco [pig], cão [dog]). This task has 15 items.

Task 4: Concepts about print. The children have to circle the correct answer from three options according to the examiner’s instruction (e.g., “Which one is a letter?” or “Which one is a word?”). This task has 10 items.

There is no time limit for any of the tasks. For each task, a total sum score is computed. Multiple responses per item and items with no response are marked as incorrect. The test materials (instructions, screening tool, and scoring) are available in Supplementary Materials. Children’s understanding of the instructions was tested in a previous pilot study. Moreover, in that study the images were discussed with the children to understand if they were clear enough.

Battery of Phonological Tests (BPF - Bateria de Provas Fonológicas; [34]). To measure phonological awareness skills we administered the “Classification of the initial syllable” subtest of the BPR. This test requires children to identify orally similar beginning syllabic sounds of words. For each of the 14 items, four images are presented to the child and the examiner speaks aloud the name of each image. Children have to indicate the two words that begin with the same syllabic sounds. In the validation study for the Portuguese population, Cronbach’s alpha for the items of this task was 0.77 [34].

Portuguese Oral Language Assessment (ALO-Avaliação da Linguagem Oral [35]). The naming subtest of the ALO was used to measure vocabulary breadth. In this test, 35 images are presented, and the child is asked to name what they see. The answers can be scored as 0, 1, or 2, according to the correction structure of the task. The maximum total score is 70. In the validation study for the Portuguese population, Cronbach’s alpha for the items of this task was 0.89 [35].

WPPSI - Wechsler Preschool and Primary Scale of Intelligence [40]. The vocabulary subtest of the WPPSI was used to assess vocabulary depth. For the first three items of this task (scored 0 or 1), children are asked to name pictures presented in a stimulus book. Afterward, 21 words are spoken aloud by the evaluator, and children are invited to define them orally. In this second part, the items can be scored as 0, 1, or 2. The maximum score possible is 45. The test is interrupted after six consecutive failed items.

Assessment Battery of Initial Reading Behaviors (BACIL - Bateria de Avaliação dos Comportamentos Iniciais de Leitura [41]). Task IV of the BACIL (concepts about print) was used to measure the children’s knowledge and conceptualizations about print. This task comprises 30 vignettes that assess print identification, print concepts, and concepts of words. For each vignette, children must identify the correct elements among four options according to the examiner’s instruction (e.g., “point to the image of a word” or “point to the first letter of the word”). In the validation study for the Portuguese population, Cronbach’s alpha for the items of this task was 0.95 [41].

Letter Recognition Task. A task was purposely created for the present study to assess alphabet knowledge. In this task, the test examiner asked children to name the 23 uppercase letters of the alphabet, presented in random order. The Cronbach’s alpha for the current study was 0.89.

Word Recognition Test (PRP—Prova de Reconhecimento de Palavras, [42]). The PRP comprises 3 training items and 40 experimental items. Each item is composed of one image and four stimulus words, out of which only one corresponds to the image. Students must observe each image and choose the corresponding word by flagging it. The PRP has a time limit of four minutes for first-grade students. It can be administered individually or in groups. In the validation study for the Portuguese population, Cronbach’s alpha for the items of this task was 0.96 [42].

Word Writing Task. We developed a word-writing task for this study. In this task, 30 words were dictated to the students, and they are asked to write them correctly. The list included regular, inconsistent, frequent, and less frequent words. The total score is the number of words written correctly.

Academic achievement in the subject Portuguese (grades). For children who were in the first grade, children’s academic grades in Portuguese, reflecting performance in this subject (including oral language and reading and writing performance), were collected at the end of the school year. These grades are expressed on a scale ranging from 1 (poor) to 4 (very good).

Teacher ratings of student reading performance. Ratings were collected from teachers at the end of the school year. The teachers were asked to rate each student on four domains—syllable recognition, word recognition, word writing, and oral comprehension—considering students’ performances across the school year. The ratings were expressed on a scale ranging from 1 (poor) to 5 (excellent).

### 2.3. Statistical Analyses

The scores in the screening tool were analyzed using Rasch model analysis, carried out using Winsteps Version 3.61.1 [43]. In the Rasch model, a difficulty parameter for each item (βi) and an ability parameter for each person (θp) are estimated and placed on a single logit scale or continuum. On this continuum, the more distant a person’s ability is from the item’s difficulty, with a higher value for the person’s ability, the higher the probability of the person correctly responding to the item and vice versa [44,45]. Therefore, the values of these two parameters can be used to check the appropriateness of the measure’s difficulty for the target group. Item fit was assessed by analyzing the mean square (MNSQ) infit and outfit Rasch statistics. Values between 0.5 and 1.5 indicate a good fit [46], but values higher than 2.0 suggest a severe misfit [47]. Reliability was also checked using two Rasch model coefficients: item separation reliability (ISR) and person separation reliability (PSR). ISR is an estimate of how likely it is to achieve the same ranking of the items in the measured variable given a different sample of comparable ability, and PSR is an estimate of how likely it is to achieve the same ordering of the people if they were given another set of items that measured the same construct [46,48]. The Kuder–Richardson formula 20 (KR-20) was also calculated as a measure of the internal consistency of the items’ scores. All three reliability coefficients range between 0 and 1, and a minimum of 0.70 is recommended.

In the second part, descriptive statistics were computed and differences in the total scores among the three groups (pre-K, kindergarten, and first grade) were tested. Gender differences were also tested. Analyses of variance (ANOVAs) were used to test for these differences, taking the results in each task of the screening tool as dependent variables and the schooling group and gender as independent variables. The skewness and kurtosis values were below |2| for all dependent variables, suggesting no severe violation of the normal distribution [49]. Partial eta squared (η2) was used as a measure of effect size: values > 0.14 indicate a large effect; >0.06, a medium effect, >0.01, a small effect [50]. To assess the evidence of validity based on relationships with other variables, the scores in each task of the screening test were correlated with the external criteria. Specifically, Pearson’s correlation coefficients were calculated. Cohen’s guidelines were used to analyze the size of the correlations: 0.10 indicates a small effect, 0.30 a medium effect, and 0.50 a large effect [51].

## 3. Results

### 3.1. Rasch Model Item Analyses and Reliability

**Figure 1:**
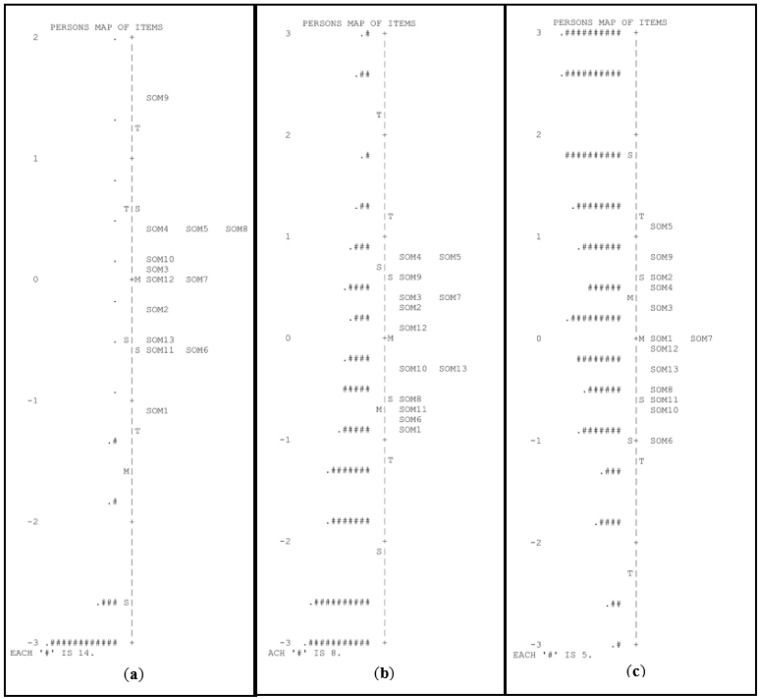
Person–item maps for the task of phonological awareness of the initial syllable in pre-K (**a**), kindergarten (**b**), and first grade of primary school (**c**). # Refers to the number of persons.

Table 2 presents the fit indices and reliability indices for the Rasch model in each group and task. The fit indices for the items (infit and outfit) were within the acceptable range. PSR indicated that the reliability of Task 1 (phonological awareness of the initial syllable) was very low for the pre-K group. An inspection of the person–item map (Figure 1) suggests that this task was extremely difficult for this group, with the mean theta being more than 1.5 logits below the mean difficulty of the items.

**Figure 2:**
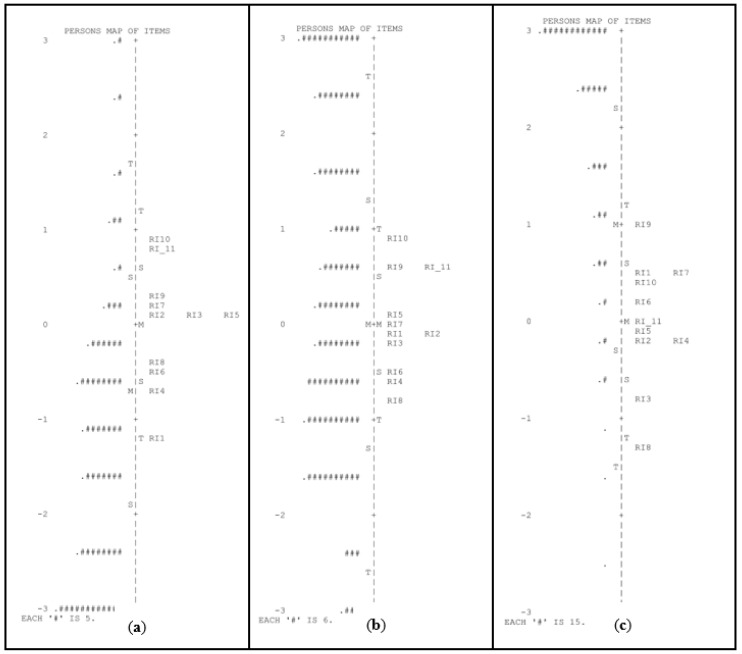
Person–item maps for the task of phonological awareness of the final syllable in pre-K (**a**), kindergarten (**b**), and first grade of primary school (**c**). # Refers to the number of persons.

For Task 2 (phonological awareness of the final syllable), the PSR value was particularly low for the primary school group (see Table 2). The person–item map presented in Figure 2 indicates that the items that compose this task had a very low difficulty for this group.

**Figure 3:**
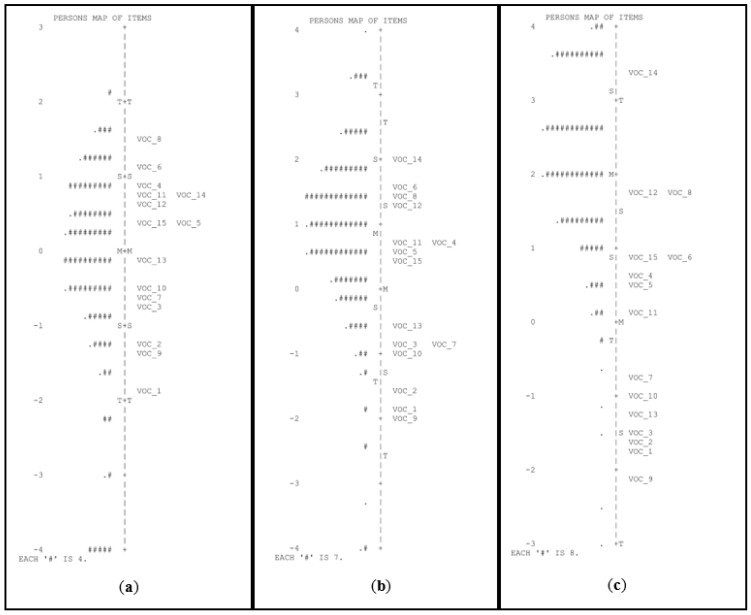
Person-item maps for the task of vocabulary in pre-K (**a**), kindergarten (**b**), and first grade of primary school (**c**). # Refers to the number of persons.

Although the PSR value fell below the threshold of 0.70 for the pre-K group (PSR = 0.61), the mean difficulty level of the items was more adequate for the ability of the sample. The vocabulary task had adequate difficulty and reliability for the pre-K and kindergarten groups (see Figure 3 and Table 2) but had low reliability and very low difficulty for the primary school group.

**Figure 4:**
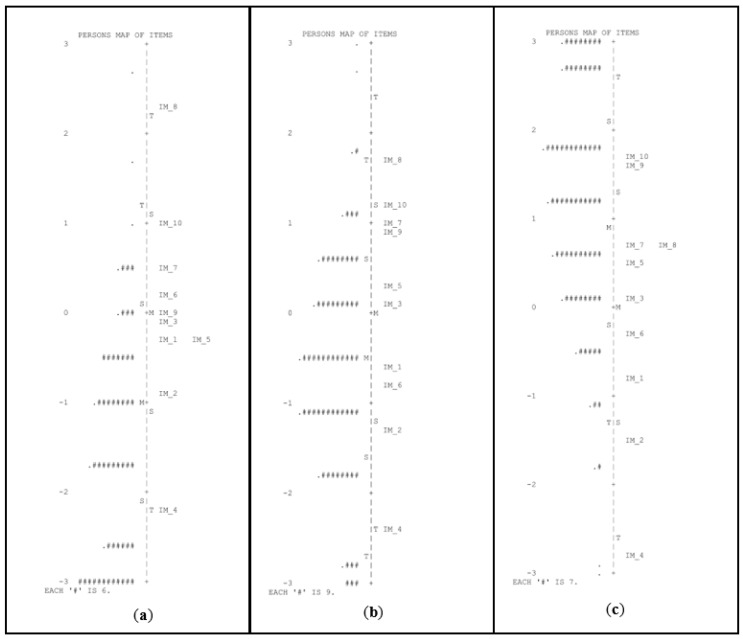
Person–item maps for the task of concepts about print in pre-K (**a**), kindergarten (**b**), and first grade of primary school (**c**). # Refers to the number of persons.

For concepts about print, PSR and KR-20 values were below 0.70 for all groups, indicating low reliability, with particularly low values for the pre-K group. An inspection of the person–item maps (see Figure 4) indicated that the task was also particularly difficult for the pre-K group.

### 3.2. Evidence of Validity Based on the Relationship with Other Variables

Table 3 presents the descriptive statistics for each measure used in this study as a function of age group. For Task 1 of the screening test (phonological awareness of the initial syllable), the ANOVA indicated no significant gender differences, F (1, 1232) = 0.971, *p* = 0.325, η2 = 0.001, nor a significant group × gender interaction, F (2, 1232) = 0.028, *p* = 0.972, η2 = 0.000. However, there was a large main effect of age group, F (2, 1232) = 362.867, *p* < 0.001, η2 = 0.371. Bonferroni post-hoc tests suggested significant differences (*p* < 0.001) between all three groups, with the phonological awareness of the initial syllable increasing sharply from an average of one correct response in pre-K, to four correct responses in kindergarten, to eight at the beginning of first grade (see Table 3).

We obtained similar results for Task 2 (phonological awareness of the final syllable). Both the main effect of gender, F (1, 1232) = 1.244, *p* = 0.265, η2 = 0.001, and gender × group interaction, F (2, 1232) = 0.877, *p* = 0.416, η2 = 0.001, were not significant. However, there was a strong significant main effect of age group, F (2, 1232) = 315.448, *p* < 0.001, η2 = 0.339, as seen by sharply increasing scores from pre-K to first grade (see Table 3).

For Task 3 (the vocabulary task), there were also no significant gender, F (1, 1232) = 3.057, *p* = 0.081, η2 = 0.002, or interaction effects, F (2, 1232) = 0.739, *p* = 0.478, η2 = 0.001. There was, again, a strong group effect, F (2, 1232) = 243.989, *p* < 0.001, η2 = 0.284, with the number of correctly identified words being significantly higher (*p* < 0.001) as the school level increased (see Table 3).

Finally, similar findings were obtained for Task 4 (concepts about print task), with no significant gender, F (1, 1232) = 0.472, *p* = 0.492, η2 = 0.000, or interaction effects, F (2, 1232) = 0.987, *p* = 0.373, η2 = 0.002, but a strong group effect, F (2, 1232) = 434.358, *p* < 0.001, η2 = 0.414. In sum, for all screening test tasks, there were no gender effects, but they were also sensitive to the expected increase in their respective skills as the school level increased.

Table 4 presents the Pearson correlation coefficients among scores for the pre-K group. The results indicate medium-to-high positive correlations among all the scores in the four screening tasks. Concerning the associations with the external criteria, most correlations were positive but weak. The correlation between phonological awareness of the initial syllable (BPR) and concepts about print task was weakly negative. Medium positive correlations were found between scores from the vocabulary task and the two standardized measures of vocabulary depth and breadth.

Table 5 presents correlation coefficients between study variables for the kindergarten group. An inspection of this table revealed medium-to-high positive correlations among the scores from the screening tasks. Additionally, strong positive correlations were found between the two phonological awareness tasks of the screening test and the BPR phonological awareness test. The vocabulary task of the screening test had a strong positive correlation with the standardized measure of vocabulary depth, and the concepts about print task of the screening test had a medium-sized correlation with the scores in the BACIL concepts about print test. The remaining correlations were mostly medium-sized (see Table 5).

Table 6 presents correlations between the variables measured alongside the screening test for the first grade group. The results suggest that the correlations among screening tasks were also medium-to-high for this group. Both phonological awareness tasks from the screening test were highly correlated with scores from the BPR phonological awareness test. The vocabulary task from the screening test had medium-to-high correlations with scores from the two standardized measures of vocabulary depth and breadth, and a high correlation was also found between the two measures of concepts of print. Letter recognition had a strong positive correlation with scores from the concepts of print task of the screening test, and also medium-sized correlations with scores from the two phonological awareness tasks.

Table 7 presents the correlations between the scores in the screening test administered at the beginning of the school year and the Portuguese grades, teachers’ ratings, and scores in the word reading and word writing tests administered at the end of the school year. Overall, scores for the screening test had moderate positive correlations with these achievement indicators.

## 4. Discussion

This study aimed to test the difficulty and reliability of the DUCLE and to collect evidence of its validity based on its relationship with other variables. We used Rasch model analysis to assess the difficulty of each item and the ability of each person to determine the suitability of the measure for the target groups [44,45,46,47]. Reliability was also assessed using two Rasch model coefficients and a measure of the internal consistency of the items’ scores [46,47]. We found that the fit indices for the items of the four tasks were within the acceptable range. However, some tasks seemed difficult for pre-K children—specifically the phonological awareness of the initial syllable and concepts about print tasks—while others were too easy for the primary school group—namely the vocabulary and phonological awareness of the final syllable tasks. For kindergarten children, all tasks had adequate difficulty and reliability.

Analyses of variance showed that there were no gender differences in the results of each task from the screening tool. Although several studies in different countries have found gender differences in young children’s emergent literacy skills (e.g., [1,52]), in Portugal efforts have been made to overcome the gender gap through educational policies [11].

We also found that there were significant differences in the performance of each task from the screening tool between pre-K, kindergarten, and primary school children. This finding suggests that the tasks are sensitive to the expected improvement in their respective skills as school level increases. Based on this result, we suggest that this tool will be most effective if the tasks are differentiated according to the schooling group. Specifically, we suggest that the most suitable tasks for pre-K are phonological awareness of the final syllable and vocabulary tasks, while in primary school the most adequate tasks are the phonological awareness of the initial syllable and concepts about print tasks. For kindergarten, all four tasks are adequate for assessing differences in children’s performance and developing tailored interventions to promote emergent literacy.

The validity of the screening tool was tested by computing correlation coefficients between task scores and related external variables. Overall, for all three groups, we found mostly medium-to-high positive correlations between the scores on the screening test and the external criteria. This finding suggests that the DUCLE tasks, which were designed using a multitier system of support, generated valid scores that can be used to identify at-risk children in pre-K, kindergarten, and primary school.

Percentile ranks are presented in Appendix A (see Table A1, Table A2, Table A3 and Table A4) for comparison to a norm-referenced sample. In line with recent research [1,6], a criterion threshold of scores one standard deviation below the mean can be used to identify skills that are at risk. We further suggest that low scores in two or more skills simultaneously can be an indicator of cumulative risk, allowing decision-making regarding additional assessment or the need to support interventions [1,6,18]. To reduce the risk of overidentifying children in need of intervention, we propose using progress monitoring with the same measure.

One of the limitations that should be considered in future studies was that no data on the children’s socioeconomic status and parental educational levels were collected. Future research should also include divergent validity studies of the DUCLE by studying the association between the results obtained in each task and the results on a test that measures a theoretically unrelated construct. Additionally, future studies could use a longitudinal methodology and use the test scores of second-grade students on the annual tests administered by the Portuguese Ministry of Education to assess the predictive properties of the DUCLE. It maybe also relevant to understand the children’s perspective regarding the experience of being administered this screening evaluation, as well as to perform assessments across the academic year to allow a closer monitoring of children’s development. The relationship between the emergent literacy skills and home literacy practices should also be addressed in future studies.

The results of the present study provide some important insights regarding collective preschool screening in emergent literacy development. The development of this screening tool aimed to fill a gap in Portugal: the need for group emergent literacy screening tools, given that individual assessments take a significant amount of time to be administrated, leaving less time and resources available for providing intervention and support to children who need it [11]. The use of DUCLE can maximize personnel resources and minimize disruption to class time. Additionally, this tool addresses all children, not only the ones with difficulties, in three domains that are known to be early literacy predictors of reading and writing: phonological awareness, vocabulary, and print knowledge [28,30].

Concerning practical implications, this screening tool that can be used in school contexts to promote emergent literacy according to children’s development. It ensures that each child has the best opportunities for academic success as it allows the identification of children who are at risk and facilitates the adjustment of the intervention. Once the results are organized by group and age, the interpretation with educators can focus on two domains: 1) the group and the skills that need to be improved; and 2) the children with lower scores in each skill and with cumulative risk (lower scores in more than one skill). These analyses facilitate the decision-making process about the type, intensity, and frequency of intervention in the group and with individual children, instead of the same intervention in children with different performances. Concerning each context of resources and children’s results, educators and specialized technicians can plan tailored interventions, proactively intervene to increase emergent literacy skills, and promote differentiation, according to the children’s skills [6,10,14].

## 5. Conclusions

The DUCLE was created to address the need for screening emerging literacy skills in children. In Portugal, the lack of formal, well-studied collective screening instruments that have strong psychometric properties limits the ability to identify children who are performing below their reference group and track performance changes. This tool offers the opportunity for early identification and intervention in foundational reading skills, preventing the development of difficulties that worsen as reading demands increase throughout schooling [2]. If all tasks are administered, the DUCLE takes approximately 45 min to administer to a group of approximately four children and requires brief training for reliable and faithful implementation and scoring. It can be administered by professionals such as psychologists, educators, and speech therapists, and does not require any special equipment.

It should be noted that this paper-based tool may be able to address some of the challenges often encountered in implementing tiered systems of support, such as evaluations conducted by school and research personnel and a focus on the development of tier 2 and tier 3 interventions. [6]. Following recommendations from other studies (e.g., [53]), to overcome the effect of short-term memory in assessing emergent literacy skills, the screening tool uses images that children can see in the booklet to discover the target sounds or semantic constructs. However, further research and evaluation may be necessary to fully understand the potential benefits and limitations of DUCLE.

## Figures and Tables

**Table 1:** Participant characteristics.

	Gender			Age
	Female	Male	Missing Information	Mean (Std. Dev.)
	N (%)	N (%)	N (%)	
Pre-K	156 (49.7%)	154 (49.0%)	4 (1.3%)	3.86 (0.35)
Kindergarten	256 (44.2%)	270 (46.6%)	53 (9.2%)	4.87 (0.34)
First grade	199 (40.9%)	203 (41.8%)	84 (17.3%)	6.03 (0.37)

**Table 2:** Fit indices and reliability for the subtests.

	Infit		Outfit		PSR	KR-20	ISR
	M (DP)	Min–Max	M (DP)	Min–Max			
Phonol. Awareness— Initial Syllable							
Pre-k	1.00 (0.15)	0.69–1.20	0.94 (0.26)	0.55–1.32	0.40	0.83	0.84
Kindergarten	1.00 (0.11)	0.87–1.26	0.99 (0.21)	0.75–1.49	0.69	0.86	0.96
First grade	1.00 (0.12)	0.82–1.27	1.01 (0.18)	0.71–1.42	0.72	0.84	0.96
Total sample	1.00 (0.14)	0.81–1.34	1.01 (0.25)	0.72–1.63	0.73	0.90	0.98
Phonol. Awareness— Final Syllable							
Pre-k	1.01 (0.12)	0.84–1.24	0.98 (0.18)	0.74–1.35	0.61	0.81	0.93
Kindergarten	1.00 (0.13)	0.86–1.31	1.00 (0.19)	0.79–1.43	0.70	0.83	0.96
First grade	1.00 (0.12)	0.85–1.25	0.98 (0.21)	0.70–1.47	0.38	0.83	0.94
Total sample	1.00 (0.14)	0.86–1.28	1.00 (0.21)	0.79–1.48	0.71	0.88	0.97
Vocabulary							
Pre-k	1.01 (0.08)	0.86–1.13	1.00 (0.13)	0.77–1.25	0.72	0.73	0.98
Kindergarten	1.00 (0.07)	0.80–1.09	0.96 (0.15)	0.53–1.11	0.71	0.71	0.99
First grade	0.99 (0.07)	0.93–1.18	0.88 (0.23)	0.44–1.42	0.51	0.62	0.99
Total sample	1.00 (0.10)	0.84–1.29	0.94 (0.20)	0.52–1.45	0.75	0.78	1.00
Concepts about print							
Pre-k	0.99 (0.10)	0.89–1.19	0.96 (0.18)	0.67–1.30	0.46	0.67	0.98
Kindergarten	1.00 (0.09)	0.89–1.20	1.01 (0.14)	0.82–1.24	0.56	0.56	0.99
First grade	0.99 (0.07)	0.89–1.11	1.00 (0.12)	0.82–1.21	0.56	0.66	0.99
Total sample	0.99 (0.09)	0.83–1.19	1.00 (0.16)	0.73–1.28	0.71	0.77	1.00

**Table 3:** Descriptive statistics.

	Pre-K		Kindergarten		First Grade	
	Mean (Std. Dev.)	Min–Max	Mean (Std. Dev.)	Min–Max	Mean (Std. Dev.)	Min–Max
Screening test						
PA—initial syllable	1.32 (2.24)	0–13	4.23 (3.68)	0–13	7.98 (3.63)	0–13
PA—final syllable	3.35 (2.91)	0–11	5.97 (3.27)	0–11	8.79 (2.67)	0–11
Vocabulary	7.05 (3.23)	0–13	9.35 (2.92)	0–15	11.81 (2.18)	0–15
Concepts about print	2.54 (2.08)	0–9	3.92 (1.99)	0–10	6.81 (2.16)	0–10
External criteria						
PA—initial syllable (BPR)	1.52 (1.43)	0–5	5.53 (3.35)	0–13	7.90 (3.81)	0–14
Vocabulary breadth (ALO)	29.62 (10.39)	10–53	38.42 (7.69)	17–53	46.00 (13.84)	10–69
Vocabulary depth (WPPSI)	17.90 (6.75)	5–33	24.35 (6.21)	7–35	28.23 (7.90)	3–41
Concepts about print (BACIL)	5.29 (3.45)	0–11	12.59 (5.68)	0–23	21.00 (7.03)	3–30
Letter recognition	-	-	-	-	13.38 (7.37)	1–26
Word reading	-	-	-	-	18.28 (9.21)	0–40
Word writing	-	-	-	-	27.45 (9.63)	0–40
Grades in Portuguese	-	-	-	-	3.18 (0.92)	1–4
TR-Syllable recognition	-	-	-	-	3.93 (1.20)	1–5
TR-Word recognition	-	-	-	-	3.81 (1.26)	1–5
TR-Oral comprehension	-	-	-	-	3.89 (1.17)	1–5
TR-Word writing	-	-	-	-	3.55 (1.24)	1–5

Note: TR = teachers’ Ratings.

**Table 4:** Correlation matrix depicting correlations between all study variables (pre-K group).

	1.	2.	3.	4.	5.	6.	7.	8.
1. Phonol. Awaren.—Initial Syllable	1	0.663 ***	0.324 ***	0.427 ***	0.295	0.084	0.255	0.255
2. Phonol. Awaren.—Final Syllable		1	0.465 ***	0.456 ***	0.248	0.253	0.288	0.112
3. Vocabulary			1	0.539 ***	0.169	0.436 *	0.431 *	0.097
4. Concepts about print				1	−0.157	0.171	0.248	0.238
5. Phon. Awaren.—Initial Syllable (BPR)					1	0.290	0.498 **	0.448
6. Vocabulary breadth (ALO)						1	0.663 ***	0.064
7. Vocabulary depth (WPPSI)							1	0.349
8. Concepts about print (BACIL)								1

Note: For variables 1–4, *n* = 314. For the remaining variables, *n* = 29; *** *p* < 0.001; ** *p* < 0.01; * *p* < 0.05.

**Table 5:** Correlation matrix depicting correlations between all study variables (kindergarten group).

	1.	2.	3.	4.	5.	6.	7.	8.
1. Phonol. Awaren.—Initial Syllable	1	0.618 ***	0.401 ***	0.357 ***	0.628 ***	0.546 ***	0.426 **	0.394 **
2. Phonol. Awaren.—Final Syllable		1	0.438 ***	0.384 ***	0.680 ***	0.439 ***	0.460 ***	0.465 ***
3. Vocabulary			1	0.508 ***	0.404 **	0.501 ***	0.288 *	0.467 ***
4. Concepts about print				1	0.254	0.234	0.230	0.437 ***
5. Phon. Awaren.—Initial Syllable (BPR)					1	0.384 **	0.387 **	0.511 ***
6. Vocabulary breadth (ALO)						1	0.548 ***	0.508 ***
7. Vocabulary depth (WPPSI)							1	0.352 **
8. Concepts about print (BACIL)								1

Note: For variables 1–4, *n* = 579. For the remaining variables, *n* = 55; *** *p* < 0.001; ** *p* < 0.01; * *p* < 0.05.

**Table 6:** Correlation matrix depicting correlations between all study variables (first grade group).

	1.	2.	3.	4.	5.	6.	7.	8.	9.
1. Phonol. Awaren.—Initial Syllable	1	0.547 ***	0.361 ***	0.378 ***	0.534 ***	0.290	0.250	0.261	0.376 *
2. Phonol. Awaren.—Final Syllable		1	0.294 ***	0.372 ***	0.539 ***	0.346 *	0.264	0.384 *	0.459 **
3. Vocabulary			1	0.362 ***	0.330 *	0.623 ***	0.446 **	0.523 ***	0.285
4. Concepts about print				1	0.622 ***	0.514 ***	0.490 **	0.590 ***	0.541 ***
5. Phon. Awaren.—Initial Syllable (BPR)					1	0.434 **	0.451 **	0.655 ***	0.540 ***
6. Vocabulary breadth (ALO)						1	0.770 ***	0.697 ***	0.330 *
7. Vocabulary depth (WPPSI)							1	0.618 ***	0.308
8. Concepts about print (BACIL)								1	0.492 **
9. Letter recognition									1

Note: For variables 1–4, *n* = 486. For the remaining variables, *n* = 40; *** *p* < 0.001; ** *p* < 0.01; * *p* < 0.05.

**Table 7:** Correlations between screening test scores and reading, writing, school achievement, and teachers’ rating for the first-grade group.

	Word Reading (*n* = 399)	Word Writing (*n* = 399)	Grades in Portuguese (*n* = 284)	Teachers’ ratings (N = 387)				
				Syllable Recognition	Word Recognition	Oral Comprehension	Word Writing	Syllable Recognition
1. Phonol. Awaren.—Initial Syllable	0.324 ***	0.380 ***	0.438 ***	0.409 ***	0.397 ***	0.418 ***	0.431 ***	0.324 ***
2. Phonol. Awaren.—Final Syllable	0.282 ***	0.481 ***	0.465 ***	0.406 ***	0.395 ***	0.373 ***	0.399 ***	0.282 ***
3. Vocabulary	0.273 ***	0.329 ***	0.368 ***	0.343 ***	0.344 ***	0.384 ***	0.346 ***	0.273 ***
4. Concepts about print	0.404 ***	0.473 ***	0.430 ***	0.473 ***	0.491 ***	0.449 ***	0.483 ***	0.404 ***

Note: *** *p* < 0.001.

## Data Availability

The raw data supporting the conclusions of this article will be made available by the authors, when requested.

## Supplementary Materials

The following supporting information can be downloaded at: https://www.mdpi.com/article/10.3390/children10020306/s1, Instructions, Screening Tool and Scoring.

## Appendix A

DUCLE’s percentile ranks.

**Table A1:** Phonological awareness of the initial syllable.

Percentiles	Kindergarten	First Grade
5	0	2
10	0	3
25	1	5
50	3	8
75	7	11
90	10	13
95	12	13

**Table A2:** Phonological awareness of the final syllable.

Percentiles	Pre-K	Kindergarten
5	0	1
10	0	2
25	1	3
50	3	6
75	5	9
90	8	11
95	10	11

**Table A3:** Vocabulary.

Percentiles	Pre-K	Kindergarten
5	0	4
10	2	6
25	5	8
50	7	10
75	10	11
90	11	13
95	12	13

**Table A4:** Concepts of print.

Percentiles	Kindergarten	First Grade
5	1	3
10	1	4
25	3	5
50	4	7
75	5	8
90	6	10
95	7	10

## References

[1] Ecalle J., Thierry X., Magnan A. (2020). A brief screening tool for literacy skills in preschool children: An item response theory analysis. J. Psychoeduc. Assess..

[2] Gutiérrez N., Jiménez J.E., de León S.C., Seoane R.C. (2020). Assessing foundational reading skills in kindergarten: A curriculum-based measurement in Spanish. J. Learn. Disabil..

[3] Dickinson D.K., McCabe A., Anastasopoulos L., Peisner-Feinberg E.S., Poe M.D. (2003). The comprehensive language approach to early literacy: The interrelationships among vocabulary, phonological sensitivity, and print knowledge among preschool-aged children. J. Educ. Psychol..

[4] Ford J.W., Kern A.M., Hosp M.K., Missall K.N., Hosp J.L. (2018). Improving efficiency for making screening decisions: A statewide comparison of early literacy curriculum-based measurement tools. Learn. Disabil. Res. Pract..

[5] National Early Literacy Panel (2008). Developing early literacy. Literacy.

[6] Stuckey A., Albritton K. (2020). Exploring the use of a multiple-gating screening process to identify preschool-age children for multitiered instructional support. Top. Early Child. Spec. Educ..

[7] Clay M. (1972). Reading. The Patterning of Complex Behaviour.

[8] Clay M. (1979). What Did I Write? Beginning Writing Behaviour.

[9] Crespo P., Jiménez J.E., Rodríguez C., Baker D., Park Y. (2018). Differences in growth reading patterns for at-risk Spanish-Monolingual children as a function of a Tier 2 intervention. Span. J. Psychol..

[10] Bailet L.L., Zettler-Greeley C., Lewis K. (2018). Psychometric profile of an experimental emergent literacy screener for preschoolers. Sch. Psychol. Q..

[11] Hendricks A.E., Adlof S.M., Alonzo C.N., Fox A.B., Hogan T.P. (2019). Identifying Children at Risk for Developmental Language Disorder Using a Brief, Whole-Classroom Screen. J. Speech Lang. Hear. Res. J. Speech Lang. Hear Res..

[12] Carvalho M., Cruz J., Azevedo H., Fonseca H. (2022). Measuring inclusive education in Portuguese schools: Adaptation and validation of a questionnaire. Front. Educ..

[13] Pereira F., Crespo A., Trindade A.R., Cosme A., Croca F., Breia G., Franco G., Azevedo H., Fonseca H., Micaelo M. (2018). Para uma Educação Inclusiva: Manual de Apoio à Prática.

[14] Fuchs L.S., Fuchs D., Fuchs D., Fuchs L.S., Vaughn S. (2008). The role of assessment within the RTI framework. Response to Intervention: A Framework for Reading Educators.

[15] Greenwood C.R., Carta J.J., Schnitz A.G., Irvin D.W., Jia F., Atwater J. (2019). Filling an Information Gap in Preschool MTSS and RTI Decision Making. Except. Child..

[16] Keller-Margulis M.A., Ochs S., Reid E.K., Faith E.L., Schanding G.T. (2019). Validity and diagnostic accuracy of early written expression screeners in kindergarten. J. Psychoeduc. Assess..

[17] Mouzaki A., Spyropoulou E., Ralli A.M., Antoniou F., Diamanti V., Papaioannou S. (2020). The dimensionality of oral language ability: Evidence from young Greek children. J. Speech Lang. Hear. Res..

[18] Sai Iyer D., Akshoomoff D., Malcarne N., Hattrup V., Berger K., Gahagan S., Needlman S.R. (2018). Development of a brief screening tool for early literacy skills in preschool children. Acad. Pediatr..

[19] Schluter P.J., Audas R., Kokaua J., McNeill B., Taylor B., Milne B., Gillon G. (2020). The efficacy of preschool developmental indicators as a screen for early primary school-based literacy interventions. Child Dev..

[20] Jenkins J.R., Schiller E., Blackorby J., Thayer S.K., Tilly W.D. (2013). Responsiveness to intervention in reading: Architecture and practices. Learn. Disabil. Q..

[21] Fuchs L.S., Vaughn S. (2012). Responsiveness-to-intervention: A decade later. J. Learn. Disabil..

[22] Meyer J.P., Invernizzi M.A., Ford K.L. (2019). Internal structure and item characteristics of the Phonological Awareness Literacy Screening in Spanish for Preschool. Assess. Eff. Interv..

[23] Jiménez J.E., Gutiérrez N., Jimenez J.E. (2019). Indicadores de Progreso de Aprendizaje en Lectura (IPAL)—Educación Infantil (5 años) [Indicators of Basic Early Reading Skills—Kindergarten]. Modelo de Respuesta a la Intervención. Un Enfoque Preventivo para el Abordaje de las Dificultades Específicas de Aprendizaje [Response to Intervention Model: A Preventive Approach for Learning Disabilities].

[24] Whitehurst G.J., Lonigan C.J. (2010). Get Ready to Read! Revised.

[25] Sénéchal M., LeFevre J. (2001). Storybook reading and parent teaching: Links to language and literacy development. New Dir. Child Adolesc. Behav..

[26] Viana F.L., Cruz J., Ribeiro I., Santos M.J., Barrera S.D. (2019). Para um olhar positivo sobre o papel da família na literacia familiar. Aprender a ler e a Escrever. Bases Cognitivas e Práticas Pedagógicas.

[27] Cruz J. (2011). Práticas de Literacia Familiar e o Desenvolvimento Literácito das Crianças. [Tese de Doutoramento não Publicada].

[28] Castro D.A.S., Barrera S.D. (2019). The contribution of emergent literacy skills for early reading and writing achievement. Trends Psychol..

[29] Cabell S.Q., Justice L.M., Logan J.A.R., Konold T.R. (2013). Emergent literacy profiles among prekindergarten children from low-SES backgrounds: Longitudinal considerations. Early Child. Res. Q..

[30] Kim Y.-S., Petscher Y. (2011). Relations of emergent literacy skill development with conventional literacy skill development in Korean. Read. Writ..

[31] Albritton K., Terry N., Truscott S. (2018). Examining the effects of performance feedback on preschool teachers’ fidelity of implementation of a small-group phonological awareness intervention. Read. Writ. Q..

[32] Goswami U., Neuman S.B., Dickinson D.K. (2001). Early phonological development and the acquisition of literacy. Handbook of Early Literacy Research.

[33] Storch S.A., Whitehurst G.J. (2002). Oral language and code-related precursors to reading: Evidence from a longitudinal structural model. Dev. Psychol..

[34] Silva C. (2003). Até à Compreensão do Princípio Alfabético: A Interacção Entre a Evolução das Conceptualizações Infantis Sobre a Escrita e os Progressos na Consciência Fonológica—Três Estudos Experimentais.

[35] Sim-Sim I. (2004). Avaliação da Linguagem Oral.

[36] Sapage S.P., Cruz-Santos A. (2021). Portuguese Early Literacy Screening Tool-RaLEPE: A pilot study. Rev. De Investig. En Logop..

[37] Batalha J., Lobo M., Estrela A., Bragança B. (2021). Avaliação da linguagem oral e escrita no pré-escolar e nos primeiros anos de escolaridade. Rev. Da Assoc. Port. De Linguística.

[38] Silva I., Marques L., Mata L., Rosa M., Orientações Curriculares para a Educação Pré-Escolar (2016). Ministério da Educação (DGE). http://www.dge.mec.pt/ocepe/sites/default/files/Orientacoes_Curriculares.pdf.

[39] Cardona M.J., Silva I.L., Marques L., Rodrigues P., Planear e Avaliar na Educação Pré-Escolar (2021). Ministério da Educação/Direção-Geral da Educação (DGE). https://www.dge.mec.pt/sites/default/files/EInfancia/documentos/planearavaliar.pdf.

[40] Wechsler D. (2003). Escala de Inteligência de Wechsler para a Idade Pré-Escolar e Primária—Edição Revista (WPPSI-R): Manual. [Wechsler Preschool and Primary Scale of Intelligence—Revised (WPPSI-R): Portuguese Manual].

[41] Teixeira M. (1993). Comportamentos Emergentes de Leitura: Aspectos Cognitivos e Linguísticos.

[42] Viana F.L., Ribeiro I. (2010). A PRP—Prova de Reconhecimento de Palavras.

[43] Linacre J.M., Wright B.D. (2001). Winsteps, Version 3.61.1.

[44] Cadime I., Ribeiro I., Viana F.L., Santos S., Prieto G. (2014). Calibration of a reading comprehension test for Portuguese students. An. De Psicol..

[45] Santos S., Cadime I., Viana F.L., Prieto G., Chaves-Sousa S., Spinillo A.G., Ribeiro I. (2016). An application of the Rasch model to reading comprehension measurement. Psicol. Reflexão E Crítica.

[46] Gómez L.E., Arias B., Verdugo M.A., Navas P. (2012). Application of the Rasch rating scale model to the assessment of quality of life of persons with intellectual disability. J. Intellect. Dev. Disabil..

[47] Linacre J.M. (2002). What do infit and outfit, mean-square and standardized mean?. Rasch Meas. Trans..

[48] Bond T.G., Fox C.M. (2007). Applying the Rasch Model: Fundamental Measurement in the Human Sciences.

[49] Hair J.F., Black W.C., Babin B.J., Anderson R.E. (2009). Multivariate Data Analysis.

[50] Cohen J. (1988). Statistical Power Analysis for the Behavioral Sciences.

[51] Cohen J. (1992). A power primer. Psychol. Bull..

[52] Deasley S., Evans M.A., Nowak S., Willoughby D. (2018). Sex Differences in Emergent Literacy and Reading Behaviour in Junior Kindergarten. Can. J. Sch. Psychol..

[53] Sim-Sim I., Silva A., Nunes C. (2008). Linguagem e Comunicação no Jardim-de-Infância: Textos de Apoio para Educadores de Infância.

