# Supplementary material for: Development of a Group Emergent Literacy Screening Tool
Source: Children (Basel). 2023 Feb 6;10(2):306. doi: 10.3390/children10020306 (PMC9955426; doi:10.3390/children10020306)
Supplement: Supplementary file 1 [file children-10-00306-s001.zip › children-2148714-supplementary.pdf]

INSTRUÇÕES_DUCLE

Descobrir o som inicial

Neste jogo vão ouvir quatro palavras. Destas 4 palavras têm de encontrar duas palavras que comecem pelo mesmo som inicial.

Exemplo 1: Temos as imagens: vaca, mola, lata, lápis. Repitam vocês.
Quais destas imagens começam com o mesmo som inicial? (Podem salientar o som inicial para chamar atenção das crianças). Lata e lápis começam com o mesmo som. Então, fazemos uma roda à volta de lata e de lápis.

Exemplo 2: Temos as imagens: bola, rato, flores, raquete. Repitam vocês.
Quais destas imagens começam com o mesmo som inicial? (Podem salientar o som inicial para chamar atenção das crianças). Rato e raquete começam com o mesmo som. Então, fazemos uma roda à volta de rato e de raquete.

Para começar o jogo temos todos de ficar em silêncio. Mesmo quando descobrirem as imagens corretas, não podem falar. Vamos começar!

1. Agora colocam o dedo no número 1. Temos as imagens: saco, garfo, cadeira, sapo. Repitam vocês. Quais destas imagens começam com o mesmo som inicial? Façam uma roda à volta das duas imagens que começam com o mesmo som.
2. Agora colocam o dedo no número 2. Temos as imagens: alface, torrada, tomate, barco. Repitam vocês. Quais destas imagens começam com o mesmo som inicial? Façam uma roda à volta das duas imagens que começam com o mesmo som.
3. Agora colocam o dedo no número 3. Temos as imagens: copo, bola, sapato, bota. Repitam vocês. Quais destas imagens começam com o mesmo som inicial? Façam uma roda à volta das duas imagens que começam com o mesmo som.
4. Agora colocam o dedo no número 4. Temos as imagens: garrafa, limão, cereja, livro. Repitam vocês. Quais destas imagens começam com o mesmo som inicial? Façam uma roda à volta das duas imagens que começam com o mesmo som.

5. Agora colocam o dedo no número 5. Temos as imagens: gato, coleira, árvore, coração. Repitam vocês. Quais destas imagens começam com o mesmo som inicial? Façam uma roda à volta das duas imagens que começam com o mesmo som.
6. Agora colocam o dedo no número 6. Temos as imagens: mola, casa, mota, cereja. Repitam vocês. Quais destas imagens começam com o mesmo som inicial? Façam uma roda à volta das duas imagens que começam com o mesmo som.
7. Agora colocam o dedo no número 7. Temos as imagens: porta, xilofone, colher, chinelos. Repitam vocês. Quais destas imagens começam com o mesmo som inicial? Façam uma roda à volta das duas imagens que começam com o mesmo som.
8. Agora colocam o dedo no número 8. Temos as imagens: maçã, velho, vela, cama. Repitam vocês. Quais destas imagens começam com o mesmo som inicial? Façam uma roda à volta das duas imagens que começam com o mesmo som.
9. Agora colocam o dedo no número 9. Temos as imagens: tapete, fato, saia, faca. Repitam vocês. Quais destas imagens começam com o mesmo som inicial? Façam uma roda à volta das duas imagens que começam com o mesmo som.
10. Agora colocam o dedo no número 10. Temos as imagens: doninha, bolacha, dominó, espelho. Repitam vocês. Quais destas imagens começam com o mesmo som inicial? Façam uma roda à volta das duas imagens que começam com o mesmo som.
11. Agora colocam o dedo no número 11. Temos as imagens: mapa, óculos, lua, lupa. Repitam vocês. Quais destas imagens começam com o mesmo som inicial? Façam uma roda à volta das duas imagens que começam com o mesmo som.
12. Agora colocam o dedo no número 12. Temos as imagens: nove, serra, caneta, seta. Repitam vocês. Quais destas imagens começam com o mesmo som inicial? Façam uma roda à volta das duas imagens que começam com o mesmo som.
13. Agora colocam o dedo no número 13. Temos as imagens: girassol, borracha, girafa, anel. Repitam vocês. Quais destas imagens começam com o mesmo som inicial? Façam uma roda à volta das duas imagens que começam com o mesmo som.

Onde está?

Neste jogo vão encontrar 4 imagens e vão ter de fazer uma roda na imagem que ouvirem. Por exemplo, coloquem o dedo na pinta preta e façam uma roda na vaca. Procuram nas diferentes imagens e, quando encontrarem uma vaca, fazem uma roda. Neste jogo temos de estar concentrados, por isso, temos de fazer silêncio. Vamos começar!

1. Coloquem o dedo no número 1 e façam uma roda no Relógio
2. Coloquem o dedo no número 2 e façam uma roda no Trovão
3. Coloquem o dedo no número 3 e façam uma roda no Cofre
4. Coloquem o dedo no número 4 e façam uma roda na Muralha
5. Coloquem o dedo no número 5 e façam uma roda no Retrato
6. Coloquem o dedo no número 6 e façam uma roda no Automóvel
7. Coloquem o dedo no número 7 e façam uma roda no Cadeado
8. Coloquem o dedo no número 8 e façam uma roda no Losango
9. Coloquem o dedo no número 9 e façam uma roda na Ilha
10. Coloquem o dedo no número 10 e façam uma roda no Globo
11. Coloquem o dedo no número 11 e façam uma roda no Retângulo
12. Coloquem o dedo no número 12 e façam uma roda na Jiboia
13. Coloquem o dedo no número 13 e façam uma roda no Alfabeto
14. Coloquem o dedo no número 14 e façam uma roda na Face
15. Coloquem o dedo no número 15 e façam uma roda no Nabo

Conceitos sobre o impresso

Esta atividade é parecida com a anterior. Colocamos o dedo numa imagem e rodeamos aquilo que vos pedir.

Vamos começar?

1. Coloquem o dedo na vaca e façam uma roda na palavra.
2. Coloquem o dedo no coração e façam uma roda na letra.
3. Coloquem o dedo no livro e façam uma roda frase.
4. Coloquem o dedo na árvore e façam uma roda no número.
5. Coloquem o dedo na casa e façam uma roda na letra maiúscula.
6. Coloquem o dedo na mota e façam uma roda na letra minúscula.
7. Coloquem o dedo na maçã e façam uma roda na primeira palavra da frase.
8. Coloquem o dedo no rato e façam uma roda na última palavra da frase.
9. Onde está a bola, façam uma roda onde se começa a ler.
10. Onde está o sapo, façam uma roda onde termina a leitura.

Descobrir palavras que rimam

Neste jogo vão ouvir uma palavra e têm de encontrar outra palavra que rime, ou seja que acabe com o mesmo som. Por exemplo, coloquem o dedo na pinta e pensem qual a palavra que rima com JOÃO. Será: cenoura, vaca, pião (salientar os sons finais, só no exemplo). João rima com pião. João e pião acabam com o mesmo som. Então, fazemos uma roda à volta de pião.

Para começar o jogo temos todos de ficar em silêncio. Mesmo quando descobrirem a imagem correta, não podem falar. Vamos começar!

1. Agora colocam o dedo no desenho de cachecol. Que palavra acaba com o mesmo som de cachecol? Será: luva, caracol, saco. Façam uma roda à volta da palavra que rima com cachecol.
2. Agora colocam o dedo na imagem de dança. Que palavra acaba com o mesmo som de dança? Será: vestido, sapato, trança. Façam uma roda à volta da palavra que rima com dança.
3. Agora colocam o dedo no desenho de mola. Que palavra acaba com o mesmo som de mola? Será: sumo, bola, calções. Façam uma roda à volta da palavra que rima com mola.
4. Agora colocam o dedo no desenho de trenó. Que palavra acaba com o mesmo som de trenó? Será: dominó, livro, janela. Façam uma roda à volta da palavra que rima com trenó.
5. Agora colocam o dedo no desenho de chita. Que palavra acaba com o mesmo som de chita? Será: casa, leopardo, fita. Façam uma roda à volta da palavra que rima com chita.
6. Agora colocam o dedo no desenho de canja. Que palavra acaba com o mesmo som de canja? Será: laranja, meia, cama. Façam uma roda à volta da palavra que rima com canja.
7. Agora colocam o dedo no desenho de bandeira. Que palavra acaba com o mesmo som de bandeira? Será: pera, urso, cadeira. Façam uma roda à volta da palavra que rima com bandeira.
8. Agora colocam o dedo no desenho de sol. Que palavra acaba com o mesmo som de sol? Será: bolacha, banana, girassol. Façam uma roda à volta da palavra que rima com sol.
9. Agora colocam o dedo no desenho de jardim. Que palavra acaba com o mesmo som de jardim? Será: raquete, abóbora, pudim. Façam uma roda à volta da palavra que rima com jardim.
10. Agora colocam o dedo no desenho de tangerina. Que palavra acaba com o mesmo som de tangerina? Será: caneca, bailarina, maçã. Façam uma roda à volta da palavra que rima com tangerina.
11. Agora colocam o dedo no desenho de camião. Que palavra acaba com o mesmo som de camião? Será: mota, avião, anel. Façam uma roda à volta da palavra que rima com camião.

Joana Cruz, Sofia Mendes, Sofia Marques, Diana Alves & Irene Cadime

Agrupamento de Escolas: ______________________ Escola: _______________ Grupo/Turma: ________	
Nome Criança: _____________________________________ Data de Nascimento: ___________________	

DESCOBRIR O SOM INICIAL

●	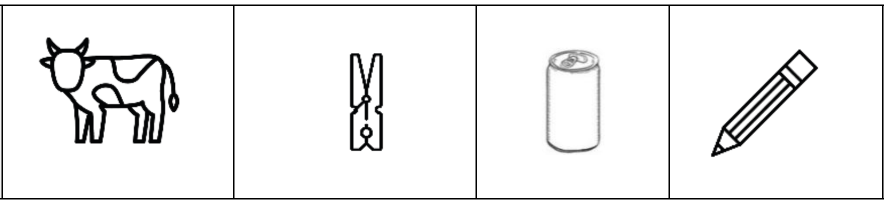	●
● ●	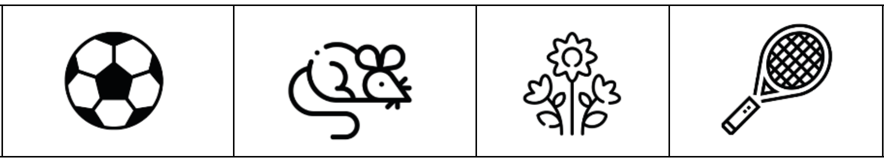	● ●
1	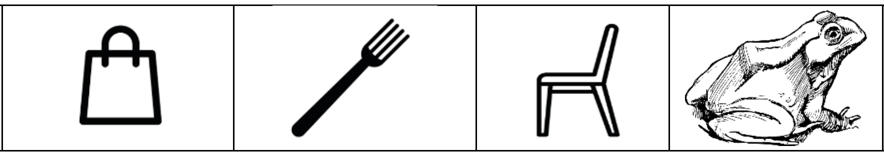	1
2	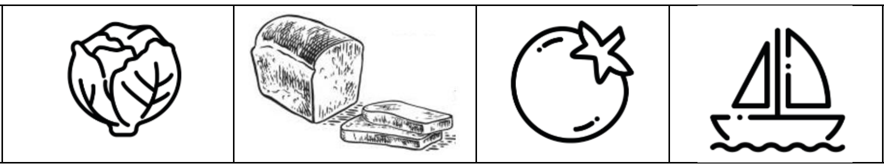	2
3	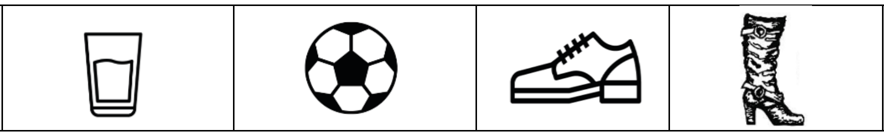	3
4	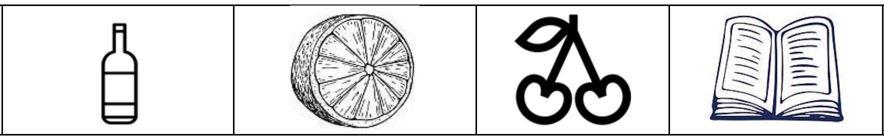	4
5	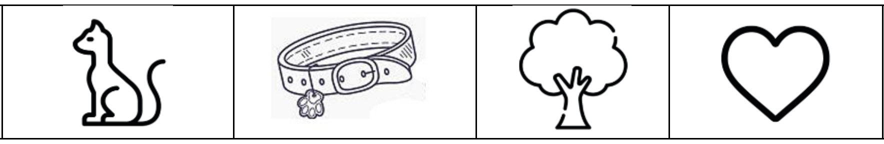	5

**6**	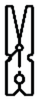	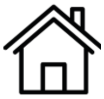	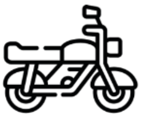	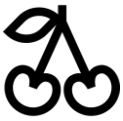	**6**
**7**	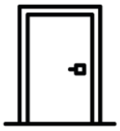	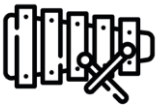	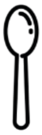	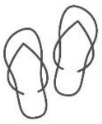	**7**
**8**	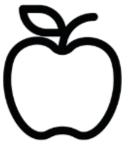	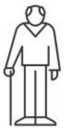	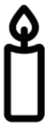	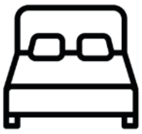	**8**
**9**	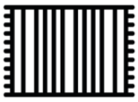	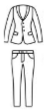	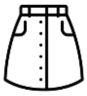	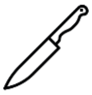	**9**
**10**	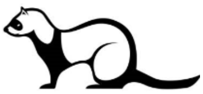	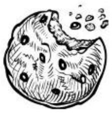	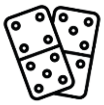	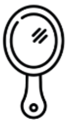	**10**
**11**	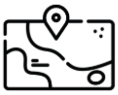	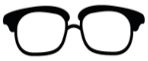	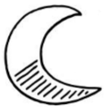	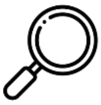	**11**
**12**	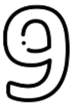	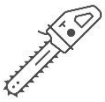	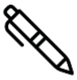	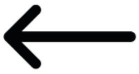	**12**
**13**	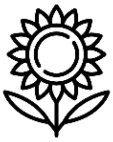	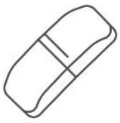	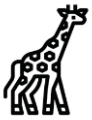	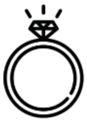	**13**

Joana Cruz, Sofia Mendes, Sofia Marques, Diana Alves & Irene Cadime

ONDE ESTÁ?

●	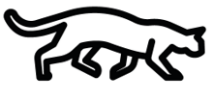	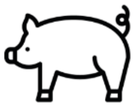	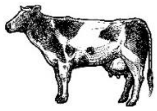	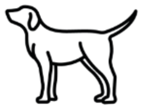	●

**1**	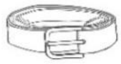	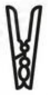	22	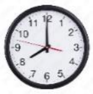	**1**
**2**	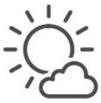	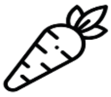	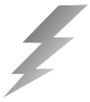	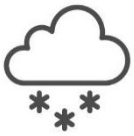	**2**
**3**	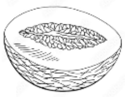	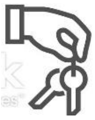	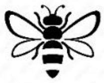	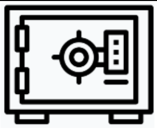	**3**
**4**	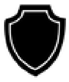	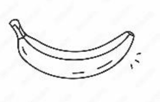	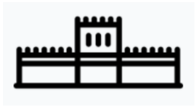	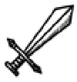	**4**
**5**	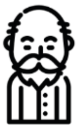	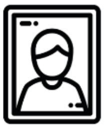	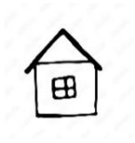	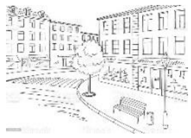	**5**
**6**	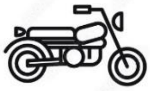	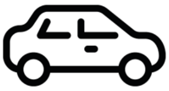	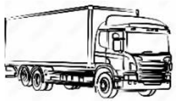	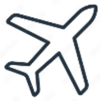	**6**
**7**	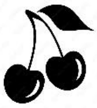	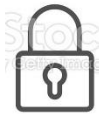	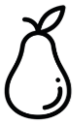	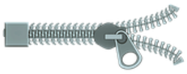	**7**

**8**	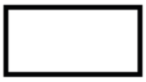	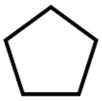	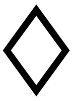	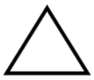	**8**
**9**	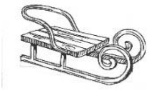	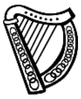	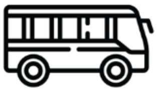	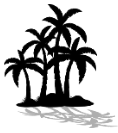	**9**
**10**	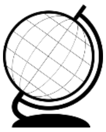	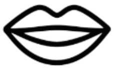	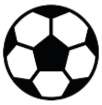	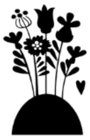	**10**
**11**	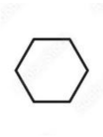	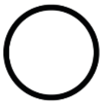	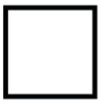	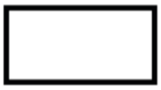	**11**
**12**	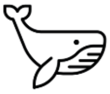	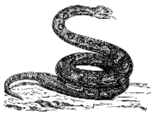	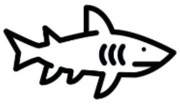	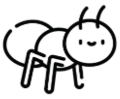	**12**
**13**	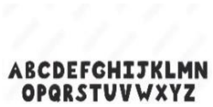	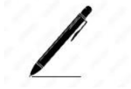	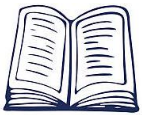	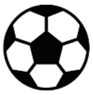	**13**
**14**	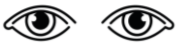	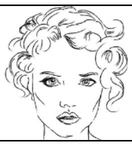	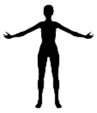	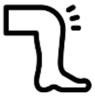	**14**
**15**	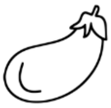	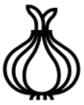	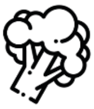	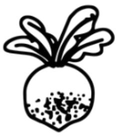	**15**

CONCEITOS SOBRE O IMPRESSO

T	viola	9
2	*	C
O cão é preto.	K	rosa
mala	8	H
a	P	Ela é alta.
L	10	t

As meninas têm olhos verdes.

O lápis é rosa.

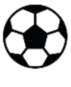

Saí de casa
para o escuro,
mas a Lua estava no céu.

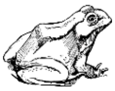

Saí de casa
para o escuro,
mas a Lua estava no céu.

Joana Cruz, Sofia Mendes, Sofia Marques, Diana Alves & Irene Cadime

DESCOBRIR PALAVRAS QUE RIMAM

●	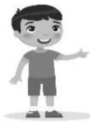	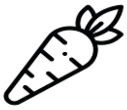	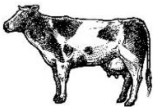	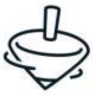	●

**1**	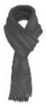	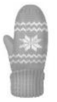	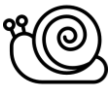	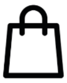	**1**
**2**	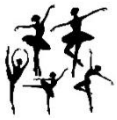	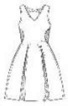	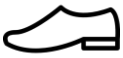	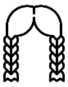	**2**
**3**	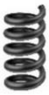	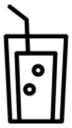	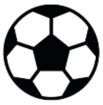	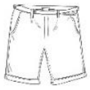	**3**
**4**	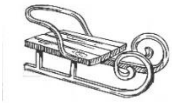	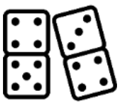	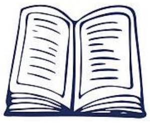	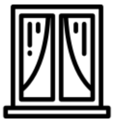	**4**
**5**	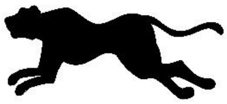	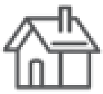	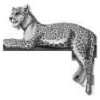	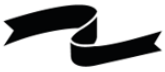	**5**
**6**	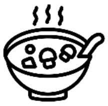	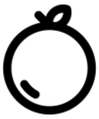	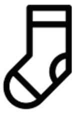	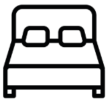	**6**
**7**	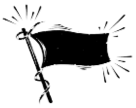	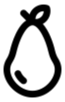	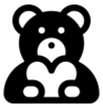	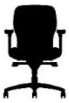	**7**

**8**	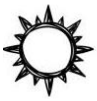	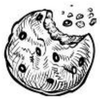	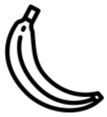	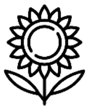	**8**
**9**	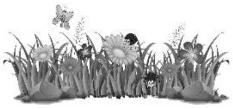	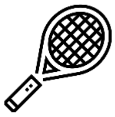	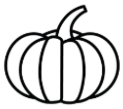	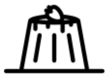	**9**
**10**	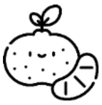	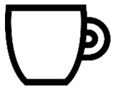	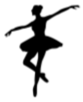	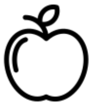	**10**
**11**	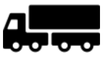	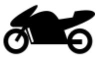	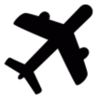	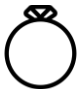	**11**

Despiste Universal de Competências de Literacia Emergente (DUCLE)

Joana Cruz, Sofia Mendes, Sofia Marques, Diana Alves & Irene Cadime

COTAÇÃO_DUCLE

Descobrir o som inicial

Cotar com 1(correto) ou 0 (incorreto). Efetuar o somatório das respostas corretas.

1. Saco, Sapo.
2. Torrada, Tomate.
3. Bola, Bota.
4. Limão, Livro.
5. Coleira, Coração.
6. Mola, Mota.
7. Xilofone, Chinelos.
8. Velho, Vela.
9. Fato, Faca.
10. Doninha, Dominó.
11. Lua, Lupa.
12. Serra, Seta.
13. Girassol, Girafa.

Onde está?

Cotar com 1(correto) ou 0 (incorreto). Efetuar o somatório das respostas corretas.

1. Relógio
2. Trovão
3. Cofre
4. Muralha
5. Retrato
6. Automóvel
7. Cadeado
8. Losango
9. Ilha
10. Globo
11. Retângulo
12. Jiboia

13. Alfabeto
14. Face
15. Nabo

Conceitos sobre o impresso

Cotar com 1(correto) ou 0 (incorreto). Efetuar o somatório das respostas corretas.

1. Palavra.
2. Letra.
3. Frase.
4. Número.
5. Maiúscula.
6. Minúscula.
7. Primeira palavra da frase.
8. Última palavra da frase.
9. Onde se começa a ler – primeira palavra ou letra.
10. Onde termina a leitura – última palavra ou letra.

Descobrir palavras que rimam

Cotar com 1(correto) ou 0 (incorreto). Efetuar o somatório das respostas corretas.

1. Caracol
2. Trança
3. Bola
4. Dominó
5. Fita
6. Laranja
7. Cadeira
8. Girassol
9. Pudim
10. Bailarina
11. Avião
